# Post-ischemic estradiol treatment reduced glial response and triggers distinct cortical and hippocampal signaling in a rat model of cerebral ischemia

**DOI:** 10.1186/1742-2094-9-157

**Published:** 2012-07-02

**Authors:** Maria Jose Pérez-Álvarez, Maria del Carmen Maza, Marta Anton, Lara Ordoñez, Francisco Wandosell

**Affiliations:** 1 Departamento de Biología (Unidad docente Fisiología Animal), Univ. Autónoma de Madrid, Madrid, 28049, Spain; 2 Centro de Biología Molecular “Severo Ochoa”, CSIC-UAM, Univ. Autónoma de Madrid, Madrid, 28049, Spain; 3 Spain and Centro de Investigación Biomédica en Red sobre Enfermedades Neurodegenerativas (CIBERNED), Madrid, Spain; 4 Centro de Biología Molecular "Severo Ochoa", CIBERNED-CSIC-UAM, Universidad Autónoma de Madrid, Cantoblanco, C/Nicolás Cabrera n° 1, Madrid, 28049, Spain

**Keywords:** MCAO, Focal ischemia, Rat, Estradiol, Brain, Estrogen, Neuroprotection, Stroke, Western blot, Immunohistochemistry, Akt

## Abstract

**Background:**

Estradiol has been shown to exert neuroprotective effects in several neurodegenerative conditions, including cerebral ischemia. The presence of this hormone prior to ischemia attenuates the damage associated with such events in a rodent model (middle cerebral artery occlusion (MCAO)), although its therapeutic value when administered post-ischemia has not been assessed. Hence, we evaluated the effects of estradiol treatment after permanent MCAO (pMCAO) was induced in rats, studying the PI3K/AKT/GSK3/β-catenin survival pathway and the activation of SAPK-JNK in two brain areas differently affected by pMCAO: the cortex and hippocampus. In addition, we analyzed the effect of estradiol on the glial response to injury.

**Methods:**

Male rats were subjected to pMCAO and estradiol (0.04 mg/kg) was administered 6, 24, and 48 h after surgery. The animals were sacrificed 6 h after the last treatment, and brain damage was evaluated by immunohistochemical quantification of ‘reactive gliosis’ using antibodies against GFAP and Iba1. In addition, Akt, phospho-Akt^Ser473^, phospho-Akt^Thr308^, GSK3, phospho-GSK3^Ser21/9^, β-catenin, SAPK-JNK, and pSAPK-JNK^Thr183/Tyr185^ levels were determined in western blots of the ipsilateral cerebral cortex and hippocampus, and regional differences in neuronal phospho-Akt expression were determined by immunohistochemistry.

**Results:**

The increases in the percentage of GFAP- (5.25-fold) and Iba1- (1.8-fold) labeled cells in the cortex and hippocampus indicate that pMCAO induced ‘reactive gliosis’. This effect was prevented by post-ischemic estradiol treatment; diminished the number of these cells to those comparable with control animals. pMCAO down-regulated the PI3K/AkT/GSK3/β-catenin survival pathway to different extents in the cortex and hippocampus, the activity of which was restored by estradiol treatment more efficiently in the cerebral cortex (the most affected region) than in the hippocampus. No changes in the phosphorylation of SAPK-JNK were observed 54 h after inducing pMCAO, whereas pMCAO did significantly decrease the phospho-Akt^Ser473^ in neurons, an effect that was reversed by estradiol.

**Conclusion:**

The present study demonstrates that post-pMCAO estradiol treatment attenuates ischemic injury in both neurons and glia, events in which the PI3K/AKT/GSK3/β-catenin pathway is at least partly involved. These findings indicate that estradiol is a potentially useful treatment to enhance recovery after human ischemic stroke.

## Introduction

Ischemic stroke is a highly disabling neurodegenerative condition with a high incidence in industrialized countries [1]. Ischemic strokes in humans account for approximately 80% of all strokes [1] and they are caused by thrombotic or embolic occlusion that decreases or suppresses the flow of blood in the middle cerebral artery (MCA), one of the main arteries supplying blood to the brain [2]. Brain injury following cerebral ischemia involves a complex succession of events that evolve spatially and temporally, causing varying degrees of cell damage depending on the characteristics of the initial insult [3]. The ischemic core and the peri-infarct zone (penumbra) suffer differing degrees of cellular damage [3-5], and it is widely accepted that cell death in the ischemic core is triggered by necrosis while that which occurs in the penumbra is predominantly mediated by apoptosis [4]. As apoptosis is a reversible process, therapeutic interventions targeting this process have the potential to prevent or limit cell death in the peri-infarct zone, even when applied post-ischemia.

MCA occlusion (MCAO) is a rodent model of ischemia that is widely used to analyze the mechanisms triggered by ischemic stroke and to study potential treatments. In this model, the cerebral cortex and the striatum are most affected brain regions, while secondary cell death occurs in the hippocampus [6]. Two types of MCAO stroke models are commonly used: transient ischemia (tMCAO) with reperfusion and permanent ischemia (pMCAO) without reperfusion. The latter is more similar to naturally occurring cerebral ischemia in humans and, thus, it is of greater clinical relevance [5]. Moreover, this model provides a useful means to test therapeutic approaches aimed at repairing the injured tissue in the late stages of cerebral ischemia [4].

Estradiol is the main female sex hormone that, in addition to its classic role in reproduction, exerts potent neurotrophic and neuroprotective effects in the brain [7]. Strokes naturally occur less in females than in age-matched males up to the age of 75 years, after which the incidence in women increases [8,9]. A large body of evidences suggests that estradiol protects against brain injury and several neurodegenerative diseases [10]. Indeed, physiological [11,12] or pharmacological levels [13] of estradiol administered prior to tMCAO or pMCAO [11,14-16] reduce the area of ischemic damage in rodent models of stroke [16-19]. While few studies have investigated the effects of post-pMCAO estradiol administration, it appears to exert a protective effect if administered within a therapeutic window of approximately 3 h post-ischemia [13,20]. However, deleterious effects of estradiol have also been described under certain conditions, possibly related to the estradiol dose or the severity and duration of ischemia, [21,22]. Moreover, some studies have demonstrated that the neuroprotective effect of estradiol is not equal in all areas affected by MCAO and that it appears to be confined to the cerebral cortex, with no detectable benefit in the striatum [10].

Three possible mechanisms have proposed for the neuroprotective effects of estrogen: (1) antioxidant effects; (2) regulation of gene transcription after binding to its classical receptor (ERα or ERβ); and (3) activation of different membrane-associated intracellular signaling pathways [10,23]. Indeed, interaction of estradiol and IGF-1 has been shown to promote neuroprotection. ERα can physically interact with downstream signaling molecules of the phosphatidylinositol 3-kinase (PI3K)/Akt/glycogen synthase kinase 3 (GSK3) pathway in an estrogen-dependent manner [23-25]. Moreover, we recently demonstrated that ERα is linked to PI3K-associated cytoplasmic signaling, and that estradiol can activate Akt/PKB and subsequently inhibit glycogen synthase kinase 3 (GSK3) [26], which may constitute a mechanism to promote neuronal survival [27]. While several studies have implicated the PI3K/Akt/GSK3 pathway in cell death after transient [28-30] or permanent [31] cerebral ischemia, it remains unclear whether modification of this pathway after pMCAO can ameliorate the effects of ischemic damage.

Mitogen-activated protein kinases (MAPKs) are divided in three families: extracellular signal regulated kinases (ERKs), c-Jun-N-terminal kinases (JNKs), and p38 MAPKs, each of which play crucial roles in signal transduction and regulate cell death and survival. MAPKs are strongly expressed in the central nervous system, and several studies have reported that alterations in MAPK expression and/or activation in post-ischemic brain tissues can affect the outcome of ischemic brain injury in animal models [32]. JNK and p38 are activated by exposing cells to stress and/or inflammatory cytokines [33]. Phosphorylation of JNK is associated with apoptosis [34], although the specific effects of JNK are highly dependent on the cell type and experimental setting [32]. Immediate activation of all three MAPKs has been described in neurons and glia in a mouse model of permanent ischemia [35], and extended activation for up to 1 day has been described for ERK and p38 [36]. The role of p-JNK, the active form of JNK, in cerebral ischemia is unclear, and the few studies that have analyzed the distribution of activated JNK following pMCAO suggest a role in neuronal apoptosis and in the angiogenic response to cerebral ischemia [37,38]. Treatment with JNK inhibitors effectively reduces the infarct volume 48 h after tMCAO in mice [39]. Furthermore, activation of the PI3K-Akt pathway in cerebellar granule neurons has been proposed to prevent neuronal cell death by suppressing JNK activation [40].

In the present study, we evaluated the impact of post-ischemic estradiol treatment in rats on the late stages of cerebral ischemia (54 h post-pMCAO), analyzing the activation of the PI3K/Akt/GSK3/β-catenin pathway and of SAPK-JNK in two brain areas affected by pMCAO: the cerebral cortex and the hippocampus. Our results indicate that estradiol treatment reduces the build-up of gliotic tissue triggered by pMCAO. Moreover, estradiol treatment reversed the down-regulation of the PI3K/Akt/GSK3/β-catenin pathway induced by pMCAO in the cortex, and to a lesser extent, in the ipsilateral hippocampus. Finally, pMCAO had no effect on the activation status of SAPK-JNK in both the cerebral cortex and hippocampus, as reflected by its phosphorylation, although post-pMCAO estradiol treatment decreases SAPK-JNK phosphorylation in both these areas, only significantly in the hippocampus.

## Materials and methods

### Animals

Experiments were performed on adult male Wistar rats (8–12 weeks old, 250–310 g) obtained from the *vivarium* at the CBM-SO. Animals were housed in a room with controlled temperature and relative humidity on an alternating 12/12 h light/dark cycle, and they were given *ad libitum* access to food and water. Animal care protocols conformed to the appropriate national legislations (decree 1201/2005, BOE no. 252) and the guidelines of the Council of European Communities (86/609/CEE).

### Induction of focal cerebral ischemia

Permanent focal cerebral ischemia was induced by occluding the middle cerebral artery using the previously described intraluminal suture method [41], with minor modifications. Rats were anesthetized with an intraperitoneal injection of a mixture of medetomidine (250 μg/kg) and ketamine (75 mg/kg), accompanied by a subcutaneous injection of atropine (100 μg/kg). The right common carotid artery (CCA) was exposed and dissected, and the right external carotid artery (ECA) and right internal carotid artery (ICA) were isolated. The first artery branches of the ECA and the pterygopalatine artery were electro-cauterized using a High Temperature Cautery Power Handle (Aaron Medical, Clearwater, USA). To occlude the origins of the MCA, a 4–0 monofilament Dafilon nylon suture (B/Braun, Aesculap AG Et. Co., Tuttlingen, Germany), the tip of which was rounded by heating and that was coated with poly-L-lysine (0.1% wt/vol in deionized water; Sigma, St Louis, MO, USA), was introduced into the ECA lumen and advanced into the ICA lumen until it met mild resistance, approximately 2.2 cm beyond the CCA bifurcation. The suture was secured with a ligature and was maintained in place until sacrifice. Sham operated rats were subjected to identical surgical procedures but no suture was inserted.

### Neurological deficit score

The neurological deficit score of each rat was measured before surgery, and 6 h after pMCAO induction, just before administration of the first estradiol dose, using a slightly modified version of the method described by Yrjänheikki [42]. To determine whether ischemia was induced correctly, the assessment was performed by an individual who was blind to the experimental treatment groups. A six-point scale of neuromotor function was graded as follows: Grade 5, normal extension of both forelimbs towards the floor when lifted; Grade 4, consistently reduced resistance to a lateral push towards the paretic side; Grade 3, circling towards the paretic side when pulled and lifted by the tail; Grade 2, circling towards the paretic side when pulled by the tail; Grade 1, spontaneous circling towards the paretic side; Grade 0: no spontaneous motion. Other neurological abnormalities were also recorded, such as alterations in balance, sensorial perception (acoustic, visual localization) and reflex responses (corneal, palpebral, postural correction).

### Estradiol administration

A total of 46 male rats were randomly distributed into the following experimental groups: sham vehicle (SV, n = 12), ischemia vehicle (IV, n = 12), sham estradiol (SE, n = 10), and ischemia estradiol (IE, n = 12). Groups SE and IE groups received three doses of 0.04 mg/kg β-estradiol (Sigma, St Louis, MO, USA, ref E8875) in a total volume of 500 μL, delivered by intra-peritoneal injection 6, 24, and 48 h after pMCAO induction. Groups IV and SV received 500 μL of the vehicle alone (1% ethanol in saline). All animals were sacrificed 6 h after the last treatment.

### Western blotting

Animals were sacrificed by exposure to CO_2_, decapitated and their brains were removed quickly. The right cerebral cortex (ischemic core) and right hippocampus (penumbral area) were dissected out and frozen at −80 °C for subsequent analysis. Tissue samples were homogenized with a Teflon-glass homogenizer in ice-cold lysis buffer containing: 20 mM Hepes [pH 7.4], 100 mM NaCl, 100 mM NaF, 5 mM EDTA, 1% Triton X-100, 1 mM Na_3_VO_4_, and a protease inhibitor cocktail (Roche Applied Science, Mannheim, Germany). Samples were kept on ice for 30 min and the insoluble material was then removed by centrifugation at 12,000 x g for 15 min. The pellet was discarded and the supernatants were analyzed in western blots. The protein concentration of each sample was determined using the DC Protein Assay kit (BioRad, Hercules, CA, USA) according to the manufacturer’s instructions. The proteins were resolved by SDS-PAGE (8%) using a Mini-Protean system (Bio-Rad) and depending on the epitope to be analyzed, 30–50 μg of total protein was loaded into each lane in loading buffer containing: 0.062 M Tris (pH 6.8), 10% glycerol, 5% β-mercaptoethanol, 7.5 mM EDTA, 2% SDS, and 0.002% bromophenol blue. Samples were heated at 100 °C for 3 min before loading. After electrophoresis the proteins were electrotransferred onto nitrocellulose membranes (Whatman, Dassel, Germany) for 1.5 h at 100 mV using an electrophoretic transfer system (Mini-Trans-blot Electrophoretic Transfer Cell). Subsequently, the membranes were blocked for 1 h with 5% non-fat powdered milk in phosphate buffered saline (PBS) containing 0.1% Tween-20 (PBS-T) and then they were incubated overnight at 4 °C with the appropriate primary antibody: Akt (1:1,000; Cell Signaling no. 9272); phospho-Akt^Ser473^ (1:500; Cell Signaling no. 9271), phospho-Akt^Thr308^ (1:1,000; clone C31E5E, Cell Signaling no. 2965), β-catenin (1:800; BD Transduction Laboratories no. 610153), β-tubulin (1:1,000; clone TUB 2.1, Sigma no. T4026), β-actin (1:1,000; clone AC-15, Sigma no. A5441), GSK3 (1:1,000; Biosource no. 44–610), phospho-GSK3^Ser21/9^ (1:1,000; Cell Signaling no. 9331), SAPK-JNK (1:1,000; Cell Signaling no. 9252), pSAPK-JNK^Thr183/Tyr185^ (1:1,000; Cell Signaling no. 9251). The membranes were washed with PBS-T and incubated for 1 h at room temperature with the corresponding secondary antibodies: peroxidase-conjugated goat anti-mouse IgG (H + L, 1:1,000; Thermo Scientific no. 32430) or goat anti-rabbit IgG-HRP (1:5,000; Santa Cruz Biotechnology no. sc-2004). Specific antibody binding was revealed using the Western Lightning-ECL chemiluminiscence system (PerkinElmer, Waltham, MA, USA), according to the manufacturer’s recommendations. Autoradiography was performed on AGFA RP2 plus films, using different exposure times depending on the primary antibody used. The films were analyzed using Quantity One software, version 4.6.0 (Bio-Rad; U-Max Powerlook 2100 XL scanner) and the results from each membrane were normalized to the β-tubulin or β-actin levels detected in the same membrane, and compared with control values (vehicle-treated sham rats). Samples from all experimental groups were processed in parallel to minimize inter-assay variation. The results represent the mean of three to five independent experiments.

### Tissue collection

Deeply anesthetized animals were transcardially perfused with ice-cold saline, 54 h after pMCAO induction, followed by 4% paraformaldehyde in 0.1 M phosphate buffer (pH 7.4). The rats’ brains were removed and post-fixed overnight at 4 °C. The following day, the brains were washed three times in PBS (15 min each), and cryoprotected in 30% sucrose in PBS at 4 °C for 48–72 h. Finally, the brains were embedded in Tissue-Tek medium (Sakura, Zoeterwoude, NL) and stored at −20 °C. Coronal cryostat sections (25-μm thick) were obtained from each brain and three tissue sections were collected onto each Superfrost Ultra Plus slides (Thermo Scientific, Braunschweig, Germany), air dried and stored at −20 °C. Sections located from +2 to −2 relative to bregma were selected for immunochemistry [43].

### Immunohistochemistry

Immunohistochemical analyses were performed in a humidified chamber. The sections were dried at room temperature for at least 1 h, washed once in PBS (10 min), permeabilized for 15 min in 0.25% triton X-100 in PBS and incubated for 30 min in blocking solution containing 0.1% Triton X-100 in PBS supplemented with 1% horse serum. The slides were incubated overnight at 4 °C with the appropriate primary antibody diluted in PBS containing 0.1% Triton X-100 and 1% horse serum: monoclonal mouse anti-GFAP (1:1,000; Clon 4A11; BD Pharmigen no. 55632); anti-Iba1 rabbit (1:500; Wako no. 019–19741); and a monoclonal rabbit anti-phospho-Akt^Ser473^ (1:100; Clon D9E, Cell Signaling no. 4060). The sections were then washed three times in PBS containing 0.1% Triton X-100, and again in PBS, and endogenous peroxidase activity was quenched by incubating for 30 min in PBS containing 1% hydrogen peroxide. The slides were subsequently incubated for 1 h at room temperature with the appropriate secondary antibody: biotin conjugated anti-mouse IgG (diluted 1:200 in PBS containing 1% horse serum) or biotin conjugated anti-rabbit IgG (diluted 1:200 in PBS containing 1% normal goat serum). After washing five times with PBS, the sections were incubated for 30 min with avidin-biotin-peroxidase complex from the Vector ABC Elite kit (Vectastain, Vector laboratories Inc., Burligame, CA, USA). The sections were then washed in PBS and incubated for 15 min with diaminobenzidine reagent FAST 3'3 diaminobenzidine tablets (Sigma, D4168). Following dehydration in a series of graded ethanol dilutions (70%, 90%, 100%), the sections were cleared with xylol and mounted using Entellan New mounting medium (Electron Microscopy Sciences, Hatfield, PA, USA). For double immunostaining experiments, following incubation with the primary antibody, the sections were incubated for 1 h with anti-rabbit Alexa 488 and anti-mouse Alexa 555 fluorescent secondary antibodies (1:500; Invitrogen). After washing three times with PBS, the slices were mounted using Fluoromount G (Southern Biotech). To determine the specificity of the immunoreactions, each batch experiment included control preparations in which the primary antibody was omitted and the incubation solution replaced with blocking solution. Slides immunostained with Iba1 or GFAP were observed under an Olympus BX61 microscope and images captured using an Olympus DP50 camera. Images from slides double-stained for Akt^Ser473^ and GFAP were captured using an LSM510 laser scanning confocal microscope coupled to an inverted Axiovert 200 M microscope (Zeiss). The intensity of GFAP and Iba1 immunostaining was quantified using free image processing software (http://fiji.sc/wiki/index.php/Fiji).

### Statistical analyses

GraphPad Prism software was used for all statistical analyses and all the data are presented as the mean ± standard error of the mean (SEM). In all cases, differences were considered statistically significant where *P <*0.05. Student’s *t*-test was used to compare differences between groups. For immunohistochemical data, the positively stained area was quantified using Fiji image processing software. The effects of and estrogen treatment on the area stained positively were compared using the Student’s *t*-test.

## Results

### Neurological status 6 h post-pMCAO

**Figure 1:**
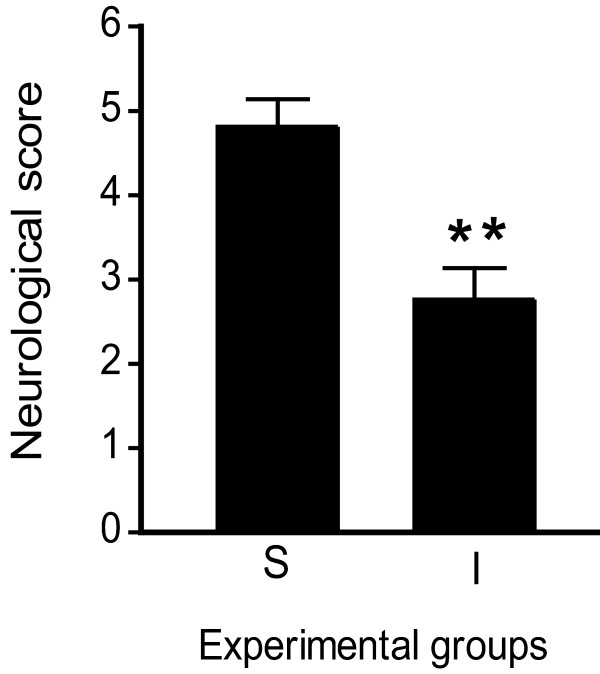
**The ischemic damage index (IDI) score decreased significantly 6 h after pMCAO induction in rats.** The IDI was measured using a neurological test, whereby 0 corresponds to the lowest degree of neurological recovery and 5 the highest. Bar graphs show the mean neurological score of all the animals studied 6 h after the onset of pMCAO. A marked reduction in the neurological score was observed in the pMCAO (IV) group when compared with the SV group (*n* = 22-24 per group; Student’s *t*-test, ** *P* ≤ 0.005). The data represent the mean ± standard error of the mean (SEM). SV, *Sham-*vehicle; IV, pMCAO-vehicle.

To confirm the induction of ischemic damage, the neurological capacity was determined in all rats before and 6 h after inducing pMCAO (Figure 1). Prior to surgery, all the rats attained the maximum score of 5 (data not shown), whereas at 6 h post-pMCAO, the sham operated animals attained an IDI score of 4.8 (± 0.3) and the ischemic rats a score of 2.7 (± 0.3). Thus, pMCAO had a significant and consistent effect on the neurological status of the rats (*P* = 0.00031), confirming their marked neurological impairment. Ischemic animals that did not attain a score of 2 to 3 were not included in the study.

### pMCAO induces reactive gliosis in the ischemic tissue, an effect attenuated by post-ischemic estradiol treatment

**Figure 2:**
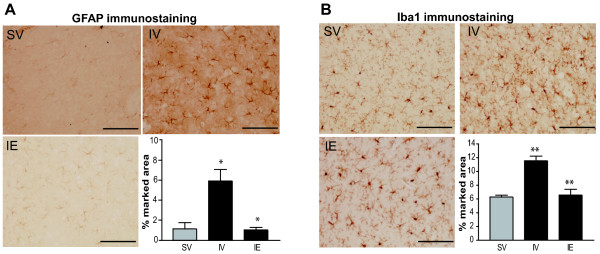
**Post-ischemic estradiol treatment attenuated ‘reactive gliosis’ in the ischemic area.** The reactive gliosis induced after 54 h of pMCAO was measured in coronal brain sections (25 μm thick) by immunohistochemisty for GFAP and Iba1, markers of astrocytes and microglia, respectively. Representative coronal sections corresponding to the injured area (bregma −1.08) of sham-vehicle (SV), pMCAO-vehicle (IV), and pMCAO-estradiol (IE) rats are shown. The accompanying bar graph shows GFAP (**A**) and Iba1 (**B**) labeling quantified in at least 12 serial coronal sections from bregma 2.04 and −1.08 with Fiji image processing software, and expressed as the percentage of labeled tissue with respect to the total area of the photographed sections. The induction of pMCAO induced an increase in the percentage area labeled for GFAP (**A**) and Iba1 (**B**) when determined 54 h after the onset of pMCAO (IV group). Post-pMCAO estradiol treatment (IE group) significantly reduced GFAP (A) and Iba1 (**B**) labeling to levels comparable with those in the sham-vehicle (SV) animals. The data represent the mean ± standard error of the mean (SEM): * P ≤ 0.05, **P ≤ 0.005; scale bar = 100 μm.

Resting glia are activated following brain damage, generating a stereotypic cellular response known as gliosis, the timing and extent of which varies depending on the initial insult [44]. To evaluate the effect of estradiol on reactive gliosis after pMCAO, we analyzed the accumulation of astrocytes and microglial cells in coronal sections of the cortex and hippocampus stained with antibodies against GFAP or Iba1. GFAP and Iba1 staining were increased in ischemic tissue, an effect that was more pronounced near the damaged area (from +2 to −2 relative to bregma). When quantified and normalized to the total tissue area, and expressed as a percentage of the damaged area, the GFAP-positive area was significantly larger in the pMCAO (IV) group (6.3 ± 2.1%) than in the corresponding area (bregma −1.08) of the SV animals (1.2 ± 1.1%, P = 0.03: Figure 2A). Similarly, the Iba1-positive area was significantly larger in the pMCAO (IV) rats (11.55 ± 1.1%) than in the corresponding area of the SV rats (6.2 ± 0.4%, P = 0.008: Figure 2B). As expected, these findings demonstrate a marked accumulation of glial cells (astrocytes and microglia) in the injured tissue 54 h after pMCAO.

Administration of estradiol after p-MCAO reduced the percentage of GFAP-positive and Iba1-positive area in damaged tissue, which fell to levels similar to those in the control animals (SV). These results indicate that post-pMCAO estradiol treatment can effectively reduce ‘reactive gliosis’ in ischemic tissue (Figure 2).

### In the cerebral cortex and hippocampus, pMCAO alters the activity of the PI3K/akt pathway but not that of JNK

**Figure 3:**
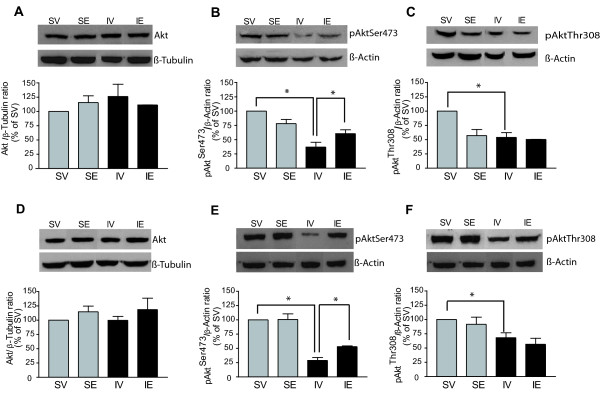
**Estradiol treatment rescued the decreased levels of phosphorylated Akt Ser-473 induced by ischemia in the cortex and hippocampus, but not the decrease in Akt Thr-308.** Akt activation was inferred in western blots of homogenates from the ipsilateral cerebral cortex (**A**, **B**, **C**) and hippocampus (**D**, **E**, **F**). The bar graphs show the levels of total Akt (**A**, **D**), pAkt Ser-473 (**B**, **E**), pAkt Thr-308 (**C**, **F**), and representative blots. The data represent the mean of five independent experiments and β-tubulin or β-actin were used as loading controls. In both the cortex and hippocampus, pMCAO (IV group) significantly reduced levels of pAKT Ser-473 (**B**, **C**) and pAkt Thr-308 (**E**, **F**) 54 h after the onset of ischemia. Post-pMCAO estradiol treatment (IE group) attenuated the decrease in pAkt Ser-473 in the cortex (**B**) and hippocampus (**E**), but had no such effect on pAkt Thr-308 in either region (**C**, **F**). No changes in total Akt levels were observed in any of the experimental groups (**A**, **D**). The data are expressed as mean ± SEM; * P ≤ 0.05; n = 6 rats per group. IE, pMCAO-estradiol; IV, pMCAO-vehicle; SE, Sham-estradiol; SV, Sham-vehicle.

In addition to its genomic action, estrogen rapidly activates cytoplasmic signaling elements like phosphoinositide 3-kinase (PI3K) [24,45]. We recently demonstrated that estradiol induces an immediate activation of Akt/PKB, and the ensuing inhibition of GSK3, in both the mouse brain and in primary cultures of neurons [26]. Thus, we investigated the role of the PI3K/Akt pathway in the protective effect of post-pMCAO estradiol administration. Cell extracts were obtained from the parietal cortical region and the ipsilateral hippocampus of treated and untreated brains, and the functional status of the PI3K pathway was evaluated with phospho-specific antibodies targeting activated or inhibited components of this pathway, as well as antibodies against the total protein. While no changes in levels of total Akt were detected in the cerebral cortex 54 h after the onset of pMCAO (IV group: Figure 3A), a clear decrease in Akt activity, was evident when Akt phosphorylation at the Ser-473 and Thr-308 residues was quantified (Figure 3B and 3 C). This reduction was more pronounced for pAkt Ser-473 (63 ± 8.4% the SV control levels; P = 0.0001) than for pAkt Thr-308 (46 ± 8.3% the SV control levels; P = 0.03). Post-pMCAO estradiol administration (IE group) partially recovered Akt activity (23.5 ± 6% increase in pAkt Ser-473 levels vs. the IV group; P = 0.03), although this effect was not observed for pAkt Thr-308 (Figure 3B and 3 C). All the quantifications of the western blots were normalized with respect to β-tubulin or β-actin.

**Figure 4:**
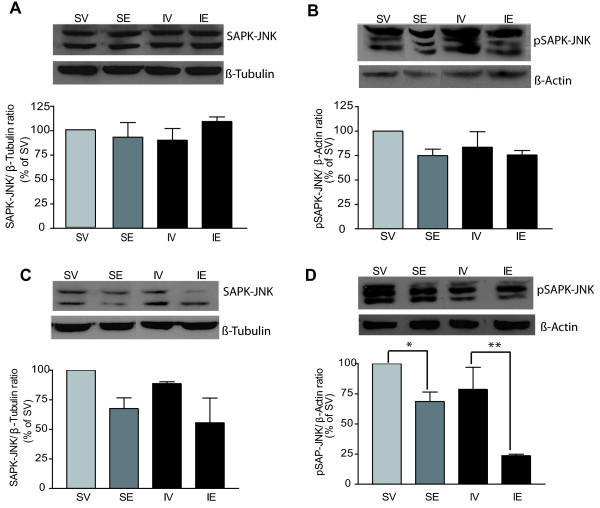
**The levels of pSAPK-JNK Thr-183/Tyr-185 were unchanged 54 h after inducing pMCAO.** The total SAPK-JNK and pSAPK-JNK Thr-183/Tyr-185 was measured in western blots of ipsilateral cerebral cortex (**A**, **B**) and hippocampus (**C**, **D**) homogenates. The bar graphs show total levels of SAPK-JNK (**A**, **C**) and pSAPK-JNK Thr-183/Tyr-185 (**B**, **D**), and representative blots. The data represent the mean of three to five independent experiments and β-tubulin or β-actin were used as loading controls. In both the cerebral cortex (**A**, **B**) or hippocampus (**C**, **D**), pMCAO had no major effect on the levels of total SAPK-JNK (**A**, **C**) or pSAPK-JNK Thr-183/Tyr-185 (**B**, **D**). Post-pMCAO estradiol treatment produced a slight decrease in pSAPK-JNK Thr-183/Tyr-185 levels in the cerebral cortex (B), and a significantly decreased in the levels of pSAPK-JNK in the hippocampus, as compared with the SV group (**D**). Data are expressed as the mean ± SEM (n = 6 rats per group). IE, pMCAO-estradiol; IV, pMCAO-vehicle; SE, Sham-estradiol; SV, Sham-vehicle.

In the hippocampus, no changes in total Akt were observed 54 h after the onset of ischemia (Figure 3D), although a significant decrease in pAkt Ser-473 was detected (72 ± 5.1% reduction vs. SV group, P = 0.005; Figure 3E) and to a lesser extent in pAkt Thr-308 (32 ± 8.5% reduction vs. SV group, P = 0.007; Figure 3F). A significant recovery in pAkt Ser-473 levels was induced by post-pMCAO estradiol treatment (IE group; 24 ± 5% increase vs. IV group, P = 0.01) but not in pAkt Thr-308 (Figure 3E and 3 F). JNK is known to contribute to apoptotic responses in many cell types. Moreover, JNK-dependent apoptotic signaling can be blocked by the activation of survival pathways, including the Akt/PKB pathway [39]. To study the effect of estradiol treatment on SAPK-JNK activation, we quantified the total and phosphorylated SAPK-JNK in the ipsilateral cortex and hippocampus 54 h after inducing pMCAO. In western blots, no changes in the cortical or hippocampal levels of phosphorylated SAPK-JNK were evident 54 h after inducing pMCAO (IV group) when compared with the SV group (Figure 4B and 4D), a measure of the activation levels of this kinase. Post-pMCAO estradiol treatment (IE group) appeared to induce a decrease in SAPK-JNK phosphorylation that was significant in hippocampus but not in cerebral cortex (Figure 4B and 4D).

**Figure 5:**
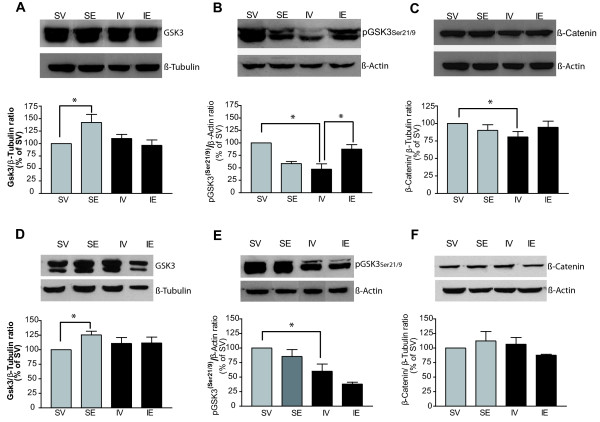
**Post-ischemic estradiol treatment restores pGSK3Ser21/9 levels in the cerebral cortex but not the hippocampus.** Levels of phosphorylated GSK3 and total β-catenin (a substrate of GSK3) were measured in western blots of homogenates from the ipsilateral cerebral cortex (**A**, **B**, **C**) and hippocampus (**D**, **E**, **F**). The bar graphs show the total GSK3 (**A**, **D**), the pGSK3Ser21/9 (**B**, **E**), and the total β-catenin (**C**, **F**), as well as representative blots. The data represent the mean of three to five independent experiments, using β-tubulin or β-actin as loading controls. In the ipsilateral cortex (**B**) and hippocampus (E), pMCAO (IV group) significantly reduced the pGSK3Ser21/9 levels and total β-catenin levels 54 h after the onset of ischemia when compared with the SV group. Post-pMCAO estradiol treatment attenuated the decrease in pGSK3Ser21/9 (B) and β-catenin (**C**) levels in the cerebral cortex, but it had no effect on pGSK3Ser21/9 levels in the hippocampus (**E**). No changes in total β-catenin levels were observed in the hippocampus in any experimental group (**F**). Total GSK3 levels increased significantly following estradiol treatment in sham operated animals (SE), both in the cortex (**A**) and hippocampus (**C**), as compared with the corresponding control (SV) group. The data are expressed as mean ± SEM: * P ≤ 0.05; n = 6 rats per group. IE, pMCAO-estradiol; IV, pMCAO-vehicle; SE, Sham-estradiol; SV, Sham-vehicle.

No changes in the total levels of SAPK-JNK were observed between experimental groups in either the cortex or hippocampus (Figure 4A and 4B). Post-pMCAO estradiol treatment alters GSK3 activity in the cerebral cortex and hippocampus. In many cell types, Akt regulates cell survival by modulating the phosphorylation of various substrates. The activity of the GSK3α/β is primarily regulated by Akt-mediated serine phosphorylation (GSK3α21/β9) and in cortical tissue GSK3 phosphorylation at residues Ser-21/9 was decreased by pMCAO (53 ± 10% reduction in IV vs. SV group, P = 0.006). Post-pMCAO estradiol treatment rescued the levels of pGSK3 Ser21/9 (40 ± 9% increase in IE vs. IV group, P = 0.01: Figure 5B) and interestingly, the levels of total GSK3α/β increased significantly in sham-operated animals after estradiol treatment (42 ± 16% increase in SE vs. SV group, P = 0.03: Figure 5A).

In the ipsilateral hippocampus pMCAO decreased the levels of pGSK3 Ser-21/9 (36 ± 5.7% decrease in IV vs. SV group), albeit to a lesser extent than that observed in the cortex (Figure 5E). Total hippocampal GSK3α/β also increased in the SE group (25 ± 6.3% greater vs. SV group, P = 0.01), yet again to a lesser extent than in the cortex (Figure 5D). However, in contrast to the effect observed in the cortex, post-pMCAO estradiol treatment did not rescue the decrease in pGSK3 Ser21/9 levels induced by ischemia in the hippocampus (Figure 5E).

GSK3β is a serine-threonine kinase that acts on a plethora of substrates, including β-catenin and the microtubule-associated protein tau [46]. β-catenin stabilization is controlled by the activity of GSK3, among other kinases [47]. Given the alterations in GSK3α/β phosphorylation provoked by estradiol treatment, we analyzed the levels of β-catenin in ischemic rats administered estradiol or the vehicle alone. In the cortex, pMCAO decreased the levels of total β-catenin (20 ± 7.5% lower in the IV vs. SV group, P = 0.03: Figure 5C), although this effect appeared to be partially, if not significantly offset by post-pMCAO estradiol treatment. In the ipsilateral hippocampi there were no changes in β-catenin levels in any of the experimental groups (Figure 5F).

### Ischemia-induced decreases in pAkt-ser 473 primarily affect neurons in the ischemic tissue

**Figure 6:**
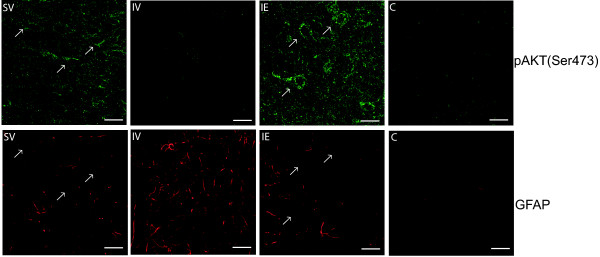
**The decrease in pAkt Ser-473 levels induced by ischemia and its subsequent rescue by estradiol treatment primarily occurs in neurons within the ischemic area.** Representative coronal brain sections corresponding to bregma −1.08 of control (SV) and pMCAO (IV) rats, and from estradiol-treated pMCAO animals (IE group). Images show the ipsilateral ischemic cortex double stained for GFAP (red) and pAkt Ser-473 (green). pMCAO (IV group) resulted in a decrease in the intensity of pAktSer-473 staining and an increase in GFAP staining intensity 54 h after the induction of ischemia, when compared with the control (IV) group. Post-pMCAO estradiol treatment rescued this decrease in pAkt Ser-473 levels and decreased GFAP staining, resulting in levels similar to those of the control (SV) group. The pAkt Ser-473 immunoreactivity (green) did not co-localize with that of GFAP (red). Representative cells exhibiting pAkt Ser-473-positive cytoplasmic labeling are indicated in SV and IE panels (arrows). The size and morphology of these cells strongly suggests they are neurons. Scale bar = 25 μm.

To determine which cell types were most affected by pMCAO-induced decrease in the Ser-473 pAkt, we performed dual-immunofluorescence experiments using specific antibodies against pAkt Ser-473 and GFAP. In the SV group, cytoplasmic anti-pAkt Ser-473 staining was predominantly located in neurons, as revealed by the distinct localization of the GFAP and pAkt Ser-473 signals (Figure 6, SV group). Following pMCAO (IV group), pAkt Ser-473 levels decreased in GFAP-negative cells (Figure 6). Post-pMCAO estradiol treatment (IE group) preferentially rescued pAkt Ser-473 levels in cells that did not express GFAP (Figure 6).

## Discussion

Ischemic stroke is the second leading cause of death worldwide, yet there are few therapeutic approaches available to treat this condition and those that exist, such as thrombolytic tissue plasminogen activator (tPA), are limited by a narrow therapeutic window [48]. Two murine models are widely used to investigate the cellular and molecular mechanisms of stroke: transient and permanent MCAO. While the onset of ischemia can be precisely defined in animal models, this is rarely the case in human patients, and indeed, the onset of symptoms may not coincide with the onset of cerebral ischemia or they may not be noticed by the patient for some time [3]. Thus, developing post-ischemia treatments represents a more realistic and clinically relevant therapeutic approach. As such, it is necessary to understand the pathophysiological events that occur in late stages of ischemia in order to develop therapeutic strategies that are effective over a broad time window following ischemia.

Previous studies demonstrated a protective effect of estradiol in pMCAO models when it was administered prior to the induction of ischemia, mimicking the circulating physiological levels of the hormone [18,19,49]. However, few studies to date have investigated the therapeutic effects of physiological estradiol treatment after the induction of ischemia. Our findings describe a potential mechanism of estrogen-induced neuroprotection in late stages of pMCAO in male rats, suggesting a significant clinical potential of estradiol to treat ischemic stroke.

Administration of estradiol (0.04 mg/kg) 6, 24, and 48 h post-pMCAO decreased the reactive gliosis 54 h post-pMCAO, as witnessed by the expression of GFAP and Iba1, an effect that was more pronounced in the ipsilateral cortex than the ipsilateral hippocampus. Differential down-regulation of the PI3K/Akt/GSK3/β-catenin pathway was observed in the cortex and hippocampus in the late stages of cerebral ischemia, while there were no changes in JNK phosphorylation following pMCAO in either region. Post-ischemic treatment with three doses of estradiol, beginning 6 h after the onset of pMCAO, partially recovered the activity of the PI3K/Akt/GSK3/β-catenin pathway, although this effect was more pronounced in the cerebral cortex (the region most affected by ischemia in this model) than in the hippocampus (secondary death cell area). Finally, the substantial decrease in pAkt (Ser-473) levels after pMCAO predominantly affected GFAP-negative cells and attending to their morphology and size, mostly neurons in the ischemic area.

Like many other neurodegenerative disorders, the reactive gliosis associated with ischemic stroke involves both astrocytes and microglia [44,50,51]. This response can vary depending on the severity and extent of brain damage, and it involves both positive elements, such as neurotrophins and anti-inflammatory components, and ‘negative elements’, including proteoglycans or components of myelin [39,51-56]. It is therefore important to consider both these aspects of the reactive glial response when developing therapies for ischemic stroke. A strong correlation between the size of the infarct area and the accumulation of microglia has been described previously in the tMCAO model [39]. However, have been describes that estrogens can either decreased [57,58], or even increased [59,60] the number of reactive astrocytes in some models of brain injury*.* The molecular causes for these differences are still unknown. Some authors postulate that this may represent the relative role of ERα and ERβ on the control of the neural inflammatory response in vivo, and it would depend on both type of injury and/or the CNS region [57]. This is, to our knowledge, the first study to analyze the effect of estradiol on the accumulation of reactive glia (both astrocytes and microglia) during cerebral ischemia processes. Indeed, post-pMCAO treatment with estradiol significantly decreased in GFAP and Iba1 immunostaining in the ischemic area (cortex), reducing their levels to those seen in the control animals (SV). The reduction in reactive gliosis following estradiol treatment demonstrates an attenuation of ischemic damage, although the mechanisms underlying this effect are poorly understood. Estradiol treatment appears to up-regulate anti-inflammatory genes in the cortex, primarily via ER alpha, and it up-regulates the synthesis of ER alpha [61]. Moreover, GSK3β inhibitors [62] attenuate the enhanced mRNA expression of proinflammatory mediators induced by ischemia in a pMCAO model, including iNOS, TNF-α and IL-1β typically released by activated glial cells.

The effect of estradiol may also be partially mediated by complementary effects in neurons. Indeed, multiple mechanisms may underlie the effects of neuroprotective agents, combining the inhibition of neuronal cell death and combating the detrimental effects of inflammation in the treatment of stroke [63]. It is widely accepted that cell death in the ischemic core is mainly triggered by necrosis, due to restricted blood flow, while that occurring in the penumbra is predominantly mediated by apoptosis [4,64,65]. As apoptosis is a reversible process, research has focused on the apoptotic pathways involved in neuronal death after MCAO, with a view to identifying important therapeutic targets. Down-regulation of the PI3K/Akt pathway, a crucial regulator of neuronal cell survival [66], has been described up to 24 h after pMCAO induction [31,67-69]. This pathway was down-regulated 54 h after the onset of pMCAO in the two tissues differentially affected by ischemia, the cerebral cortex (corresponding to the most affected region) and the ipsilateral hippocampus. Indeed, in both the ipsilateral cortex and hippocampus pMCAO induced a decrease in the levels of pAkt Ser-473 and to a lesser extent pAkt Thr-308, without significantly affecting total Akt levels, suggesting a loss of Akt activity. Phosphorylation of both Thr-308 and Ser-473 residues is required for maximum activation of Akt [70], and it is mediated by distinct kinases: phosphoinositide-dependent kinase 1 (PDK1) and mTORC2, respectively [71,72]. The mechanism of activation of mTORC2 has not been fully characterized, although its direct role in Ser473 phosphorylation has been clearly demonstrated [73]. Our findings are consistent with previous reports describing a decrease in p-PDK1(Ser^241^) and pAkt (Ser^473^) levels in the ipsilateral cortex when compared with the contralateral counterpart 24 h after of the induction of pMCAO [49]. Furthermore, we found that post-pMCAO estradiol treatment led to partial recovery of pAkt Ser-473 levels in both ipsilateral regions (cortex and hippocampus), without affecting pAkt Thr-308 levels. These results suggest that estradiol regulates mTORC2 activity without substantially altering PDK-1 activity, although the underlying mechanism remains unclear. Alternatively, pAkt Ser-473 and pAkt Thr-308 may be differentially affected by the activation of specific phosphatases.

The decrease in pAkt Ser-473 levels in pMCAO animals (IV group) was not restricted to GFAP-positive cells but rather, it occurred predominantly in neurons. Moreover, post-pMCAO estradiol treatment reversed this effect, restoring the pAkt Ser-473 levels in these cells. These findings suggest that the effect of estradiol is not limited to the attenuation of activated gliosis in glial cells but also, that it affects the activity of the PI3K/Akt survival pathway in neurons. Indeed, we recently reported that estradiol may activate or cooperate with PI3K to phosphorylate and activate Akt [23,74]. The decrease in Akt phosphorylation at both Ser473 and Thr308 residues suggested a reduction in Akt activity following pMCAO and thus, we analyzed the phosphorylation of GSK3α/β, a key downstream target of Akt. Phosphorylation of GSK3α/β by Akt at Ser-21/9 inactivates its kinase activity and may regulate cellular apoptosis [75]. After 54 h of pMCAO, the decrease in pGSK3 Ser-21/9 detected in the cortex, and to a lesser extent in the hippocampus, is consistent with the observed reduction in Akt activity and it was correlated with an increase in GSK3 activity that may promote or mediate cell death [28,76,77]. Our data demonstrate that the pMCAO-induced decrease in cortical pGSK3 Ser-21/9 is rescued by estradiol treatment, virtually reverting to the levels seen in vehicle-treated animals (SV). Based on this finding, we propose two possible mechanisms of action by which estradiol may reduce reactive gliosis after pMCAO, through: (1) the direct inhibition of GSK3 in glial cells that subsequently alters the glial response; and (2) the direct modulation of Akt, and hence the GSK3 activity in neurons, resulting in a reduced pro-inflammatory response [62]. Analysis of the ipsilateral hippocampi revealed no recovery of pGSK3 Ser-21/9 levels following estradiol treatment, suggesting that the activity of estradiol depends on the specific neurons and/or glial receptors differentially expressed in the cortex and hippocampus. Further studies will be aimed at investigating this hypothesis.

The activation of MAPK signaling pathways during ischemia plays an important role in apoptosis and inflammation. The inflammatory response increases the damage area, a process that is particularly pronounced during reperfusion [56]. In animal models of stroke several inhibitors of the JNK pathway have protective effects [39,56]. In our pMCAO model (without reperfusion), we detected no changes in total JNK or in the phosphorylation status of this enzyme at Thr183/Tyr185 residues after 54 h of pMCAO. Hence, the JNK signaling pathway does not appear to play a significant role in pMCAO, at least at 54 h after ischemia induction.

Further studies will be necessary to determine whether the neuroprotective effect of estradiol is receptor-specific, permitting the development of more selective treatments that enhance neuroprotection while avoiding some of the negative effects in non-neural cells.

## Competing interests

The authors declare that they have not competing interest.

## Authors’ contributions

FW and MJP designed the study. MJP designed and carried out the pMCAO procedure, treatments and tissue collection. MCM carried out the neurobehavioural assessment. MJP and MCM were involved in western blots experiments. MA, LO and MJP were involved in the immunohistochemistry experiments. MJP has performed the statistical analysis. FW and MJP wrote the manuscript. All authors have read and approved the final version of the manuscript.
